# Ethnic identities of immigrant and native adolescents: development and relationship to life satisfaction

**DOI:** 10.1186/s41155-018-0100-5

**Published:** 2018-07-17

**Authors:** Laura Lara

**Affiliations:** Universidad Autonoma de Chile, 5 Poniente, 1670 Talca, Chile

**Keywords:** Ethnic identity, Ethnic labels, Adolescence, Immigration, Life satisfaction

## Abstract

Ethnic identity becomes an important issue during adolescence, especially for ethnic minority groups. This study examines ethnic identities of immigrant and native adolescents who live in Spain, focusing on the ethnic labels they use, their development, and the relation with life satisfaction. Questionnaires were administered to first-generation immigrants (*n* = 501; mean age 14.6 years) as well as to their native host classmates (*n* = 501, mean age 14.3 years). Results show that ethnic identification was mainly determined by country of birth; however, in the case of immigrants, having immigrated at an early age favored the use of identity labels of the majority group. Immigrants were more likely to be in the achieved ethnic identity category than non-immigrants, although it was positively related to life satisfaction in both groups. Finally, older adolescents were not more likely to be in the achieved category than younger ones.

## Background

The development of identity is one of the most important tasks during adolescence. Social psychology perspectives (Tajfel & Turner, 1986) present ethnic identity as a social aspect of the self. From the developmental perspective, based on Erikson’s (1968) ego identity model, ethnic identity refers to the subjective sense of ethnic group membership and is conceived as a multidimensional concept that include two core dimensions (Phinney & Ong, 2007), *exploration* (process of investigating and learning more about the meaning of one’s ethnic background) and *commitment* (process of deriving a sense of membership and affective connection to ones’ ethnic group). These dimensions are similar to the *crisis* and *compromise* components used by Marcia (1966) to set the ego-identity statuses; the combination of ethnic exploration and ethnic commitment are used in the same way to establish ethnic identity statuses (Douglass & Umaña-Taylor, 2017; Yip, 2014); the four ethnic identity statuses are *foreclosed* (commitment without exploration), *achieved* (commitment with exploration), *moratorium* (exploration without commitment), and *diffused* (not engaged in exploration or commitment).

There is evidence that the construction of ethnic identity begins in a rudimentary form in childhood where children learn to identify themselves with ethnic groups, then becomes a crucial task in adolescence based on exploration of its meaning, and culminates in an achieved identity at the end of adolescence or the first years of adulthood. Thus, individuals are expected to move from ethnic identity diffusion to either foreclosure or moratorium and then to ethnic identity achievement through adolescence, based on exploration of ethnic identity that characterize the adolescence period (Umaña-Taylor et al., 2014).

Ethnic identity becomes an important component of identity especially for adolescents who have a different ethnic background from mainstream society, a situation that is beginning to be recognized as essential for the normative development of ethnic minority groups (Rivas-Drake et al., 2014). Although researchers have started to recognize the importance of the study of ethnic identity, most research has been carried out in the US society. As a result, researchers are demanding the need to study ethnic identity in other countries around the world, realizing that empirical research in the USA should not self-evidently be applied to other societies and used as the frame of reference for the field (Verkuyten, 2016). Besides identity concerns adolescents have to deal with (such as occupation, ideology, and gender roles), ethnic minority adolescents have to face other aspects derived from being a member of a group that is typically of lower status and power in society and that may be subject to discrimination (Dotterer & James, 2018).

Researchers have focused mainly on minority groups, and the results have shown that ethnic identity is more important for them compared to majority groups (Kiang & Fuligni, 2009; Xu, Farver, & Pauker, 2015). Ethnic identity seems to be a less important aspect for mainstream adolescents, who can live and work without understanding other cultural groups, while one of the challenges for minority ethnic groups is to understand mainstream culture. However, research studies have shown that ethnic identity is also important for mainstream adolescents who are settling in multicultural contexts (Adams et al., 2016).

The labels adolescents use to identify themselves are also important for ethnic development; however, they have been much less investigated (Cheon, Bayless, Wang, & Yip, 2018; Corona et al., 2017; Fuligni, Kiang, Witkow, & Baldelomar, 2008), with research that has focused on ethnic labels used by native adolescents being practically non-existing, and implies that the mainstream group has to face little ethnic threat and because of that is expected to have lower levels of identification with their ethnic groups (Adams et al., 2016). Ethnic self-identification is crucial for the development of ethnic minority groups, and specially for immigrants. As Fuligni and Tsai (2015) stated, for immigrant adolescents, it is one of the most significant challenges to face. They must learn the social categories in which host society divides people regarding ethnic identity, and at the same time, they need to figure out where they fit.

Country of birth has been found to be a crucial element for an individual’s choice of ethnic identification, immigrants who were born outside the new country (first generation) are more reluctant to abandon labels that identify themselves as members of their original countries (Fuligni & Tsai, 2015; Kiang & Witkow, 2018), whereas second-generation immigrants (who were born in a new host country) are more prone to include the new country in their self-identification. In general, labels that refer to original nationality are predominant in first-generation immigrants and are difficult to change over their life span and second-generation immigrants are more likely to use composed labels that include the country they live in together with their country of origin (Kiang, Perreira, & Fuligni, 2011; Kiang & Witkow, 2018). However, young people who arrived at the new country at early ages (before 7 years old) may use these composed labels (Fuligni et al., 2008). Moreover, those that include these concepts in their self-identification have a weaker ethnic identity than those who do not (Fuligni et al., 2008).

Finally, ethnic identity plays an important role in adolescent well-being, as life satisfaction is one of the indices that provide crucial information about immigrant adaptation. Previous research has focused on the immigrant paradox (referring to the counterintuitive results of better adaptation of first-generation immigrants compare to second and majority ethnic groups). However, this paradox has not found support outside the USA (see meta-analysis by Dimitrova, Chasiotis, & van de Vijver, 2016), and researchers highlight the need to go deep in understanding under what conditions immigrants do better (Motti-Stefanidi & Garcia Coll, 2018). One of the key aspects is ethnic identity, it has been shown that a secure and strong ethnic identity makes positive contributions to psychological well-being in ethnic minority adolescents (Manuela & Anae, 2017). An achieved ethnic identity implies an individual has a secure perception of their own ethnicity and has resolved the conflict about his/her own ethnic group, thus achieving ethnic identity becomes a source of personal strength and positive evaluation (Verkuyten, 2016).

Different research studies support these assumptions with findings suggesting a connection between ethnic identity and life satisfaction (e.g., Abubakar, Aldhafri, & Van de Vijver, 2014; Dimitrova, Johnson, & van de Vijver, 2018). However, most of the studies focus on ethnic minorities, taking for granted that ethnic identity is not an important issue for mainstream youths. As Seaton at al. (2017) pointed out, there is a dearth of empirical research that focus on ethnic identity among ethnic majority participants, including not just adolescents, but also children and adults. However, when studies include ethnic majority groups, associations between ethnic identity and life satisfaction are also reported. For example, Adams et al. (2016) found that ethnic identity was related to life satisfaction in all groups including mainstreamers, although participants were emerging adults, not adolescents.

Based on the above, this study aims to contribute to extending the knowledge of ethnic identity to other cultural contexts. The main objective of the present study is to examine ethnic identities of immigrant and native adolescents who live in Spain, focusing on the labels they use, their development, and the relation with life satisfaction.

The following hypotheses were evaluated: ethnic identification will be mainly determined by birthplace (H1); immigrant adolescents who arrived at an early age in Spain (before 7 years old) will identify themselves also with Spanish labels (H2); immigrants will have a higher rate of achieved ethnic identity than natives (H3); immigrants who do not include Spanish labels in their ethnic self-identification are more likely to have an achieved ethnic identity status (H4); older adolescents will more often have an achieved ethnic identity status than younger ones (H5); and having an achieved ethnic identity status will be positively related to life satisfaction both in immigrants and natives, with this relation being stronger in the case of immigrants (H6).

## Method

### Participants

Participants were 1002 students of compulsory secondary education in Spain, 501 were first-generation immigrants (they and both parents were born in the same country outside Spain) and 501 were native Spaniards (they and both parents were born in Spain). Criteria for participation included that they and both their parents must have been born in the same country. Participants were evenly split by gender in both groups (49.1% were girls in the immigrant group and 52.2% in the native group). The age in the sample ranged from 12 to 18 (*M* = 14.28 years, SD = 1.46, in the native group, *M* = 14.62 years, SD = 1.51, in the immigrant group). Immigrant participants were born in Latin America (67.7%, the majority of them from Ecuador, Bolivia, and Colombia), Eastern Europe (13.6%, the majority of them from Rumania), Africa (13.2%, the majority of them from Morocco), Asia (3.6%, the majority of them from China), and a 2% from the rest of Europe. They have been residing in Spain for a mean of 4.47 years (SD = 3.17) and the mean age at arrival in Spain was 10.14 (SD = 3.43).

### Measures

*Demographic variables*: A demographic questionnaire was administered to assess participants’ gender, age, country of birth and country of parents’ birth. Additionally, foreign-born adolescents were asked to report how old they were when they arrived in Spain.

*Ethnic self-identification and ethnic identity*: In an open-ended response format, participants were asked to indicate the ethnic labels that they believed best described them. Adolescents were allowed to report more than one label. We classified the immigrants’ responses based on Hutnik (1986) categories: mixed (they used both mainstream and origin group labels), assimilated (they only used mainstream group labels), ethnocentric (they only used origin group labels), and marginalized (they neither used mainstream or origin group labels). Non-immigrants’ responses were categorized into ethnocentric and marginalized. Then, they were instructed to respond to the subsequent questions in terms of the labels they chose for themselves. Ethnic identity was measured using the Spanish version (Lara & Martínez-Molina, 2016) of the Multigroup Ethnic Identity Measure-Revised (Phinney & Ong, 2007). It is a six-item scale consisting of two subscales (exploration and commitment) with three items each, rated on a 5-point Likert scale from *strongly disagree* (1) to *strongly agree* (5), such that higher scores more strongly endorse the respective constructs. Cronbach’s alphas were adequate for the whole sample (*α =* .70 for exploration, *α =* .77 for commitment, and *α =* .83 for total) as well as for separate samples of immigrants (*α =* .83 for total, *α =* .69 for exploration, and *α =* .75 for commitment) and natives (*α =* .82 for total, *α =* .69 for exploration, and *α =* .78 for commitment).

*Satisfaction with life*: the Spanish version (Atienza, Pons, Balaguer, & García-Merita, 2000) of the Satisfaction With Life Scale (Diener, Emmons, Larsen, & Griffin, 1985) was used to asses global life satisfaction rated in a Likert scale from 1 (*strongly disagree*) to 5 (*strongly agree*). Cronbach’s alphas were adequate, for the global sample (*α =* .80), as well as for immigrants (*α =* .78) and natives (*α =* .81).

### Procedure

Adolescents were recruited from 20 public high schools in the province of Seville, Spain. Upon request, the Department of Education provided a list of the schools with information about the nationality of every student. Schools that participated in this study were those with a higher percentage of immigrants. After obtaining this information, schools were contacted and asked to participate in the study. In every classroom after recruiting the foreign-born students, an equal sample of their native classmates were randomly chosen. The same researcher visited each school during school hours, gave the same instructions to participants, applied the questionnaires, and answered any questions that arose. After guaranteeing confidentiality of data and anonymity of participants, written consent was obtained from participants’ parents, as well as the adolescents own approval. All participants were given information about the study and told that participation was voluntary. Of an initial sample of 700 immigrant participants, 60 parents did not return the consent to participate in the study, 30 were excluded because their parents were born in different countries, and 10 did not read properly in Spanish and refused to participate in the study. Regarding native adolescents, they were chosen randomly between the classmates that whose parents had returned their signed consent and were willing to participate. This study was approved by the Department of Developmental Psychology at the University of Seville.

### Data analysis

Regarding ethnic identification (H1 and H2), we used Huntik categorization to determine ethnic identification and carried out descriptive analysis. To analyze the influence of age of arrival over ethnic identification, we split the sample into two groups, younger than 7 years old on arrival and 7 years old or older on arrival and carried out cross-tabulation analysis. For the following analyses, we did not include adolescents who did not identify themselves with any ethnic group (marginalized identification) because showing a clear ethnic identification was a prior requirement to establish ethnic identity.

In order to identify ethnic identity status among standardized exploration and commitment variables, K-means cluster analyses were utilized. After that, cross-tabulation analyses were conducted to examine ethnic identity differences between immigrant and natives (H3), as well as among immigrants based on their ethnic identification (H4). In order to corroborate that older adolescents are more likely to be in the achieved ethnic identity category than younger ones, we split the sample into three age groups and conducted cross-tabulation analyses to examine age differences among the identity status (H5). Finally, a factorial analysis of variance (factorial ANOVA) was conducted to test the interaction between being immigrant or native and ethnic identity status over life satisfaction (H6). Because this interaction was not significant, we conducted a single-variance analysis (ANOVA) to test the differences on life satisfaction based on ethnic identity status.

Chi-square tests were used to analyze the relationship in cross-tabulation analysis, and standardized residual analysis to know the directions of the differences. We compared the size of the standardized residuals to the critical values that correspond to an alpha of .05 (± 1.96) or an alpha of .01 (± 2.58). All statistical analyses were performed using SPSS.21.

## Results

### Ethnic identification

The majority (94.4%) of native adolescents identified themselves using Spanish labels exclusively (*ethnocentric*) and 5% did not identify with any ethnic group (*marginalized*). The remaining 0.6% identified themselves with labels that did not refer to any cultural or ethnic group and were excluded from analyses. The majority of immigrants (64.5%) were included in the *ethnocentric* category (they identified themselves exclusively with their origin group), 18.2% were categorized as *mixed* (they used both ethnic labels referring their origin group as well as the mainstream group), 9.2% as *assimilated* (they identified themselves using Spanish labels exclusively), and 5.2% as *marginalized*. Finally, 3% used labels that did not refer to any cultural or ethnical group and were excluded from analysis.

Separate cross tabulation analyses were conducted to examine differences of age at arrival to Spain on the immigrant’s ethnic identification. Chi-square results showed there was a significant relation between ethnic identification and age at arrival to Spain *χ*^2^ (3, *N* = 473) = 12.19, *p* = .007, (*V* = .16). Standardized residual indicated immigrants who arrived when they were 7 years old or older were more likely to identify themselves as ethnocentric (z = 3.3, *p* < .01) and less as assimilated (z = − 2.1, *p* < .05) and mixed (z = − 2.4, *p* < .05) compared with adolescents who arrived when they were younger.

### Ethnic identity in immigrants and natives

**Fig. 1:**
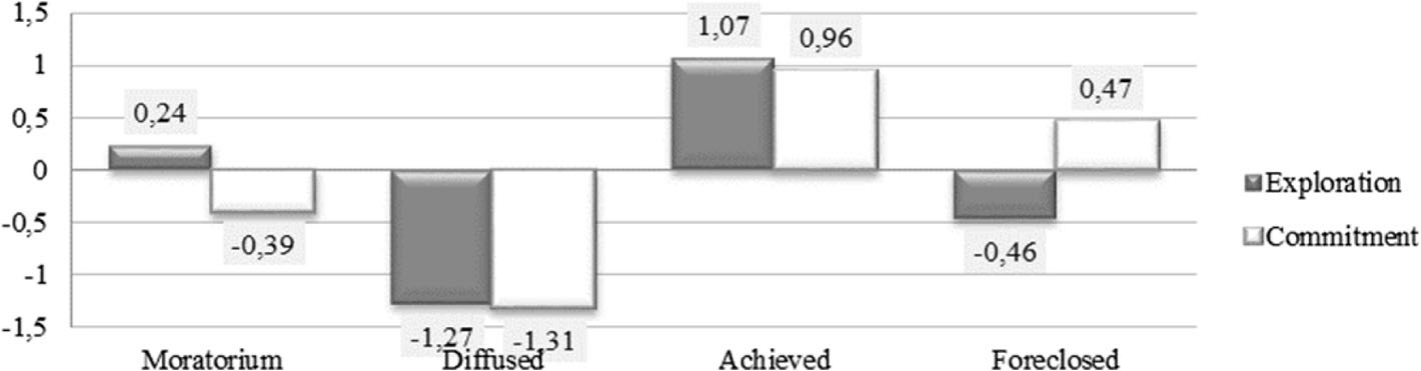
Ethnic identity status clusters

The first cluster (*n* = 260) was classified as the *moratorium* identity status, with exploration scores 0.24 standard deviations above the mean and commitment scores 0.39 standard deviation score below the mean. A second group (*n* = 159) was named *diffused* and had exploration and commitment scores around 1.3 standard deviations below the mean. A third cluster represents *achieved* identity (*n* = 273) with exploration and commitment scores around a standard deviation above the mean. The final group (*n* = 241) was classified as *foreclosed* and had exploration scores about 0.5 standard deviation below the mean, and commitment scores that were about 0.5 standard deviations above the mean (see  Fig. 1).

The percentages of immigrants and natives in each of the ethnic identity statuses were different. Immigrants were in the first place in the achieved status (36.5%), in the second place in the moratorium (28%), in the third place in foreclosed (22%), and last in diffused (13.5%). Natives were in the first place in the foreclosed status (29.6%), in the second place in moratorium (27.7%), in the third place in achieved (22%), and last in diffused (20.5%). Chi-square analysis indicated significant differences across identity status between immigrant and natives, *χ*^2^ (3, *N* = 933) = 34.11, *p* < .001, (*V* = .17). Adjusted standardized residual analyses indicated that immigrants were more likely to be in the achieved status (*z* = 4.8, *p* < .01) and less in the foreclosed (*z* = − 2.7, *p* < .01) and the diffused (*z* = − 2.9, *p* < .05) compared with natives.

Regarding differences in ethnic identity based on labels used by immigrants, chi-square results indicated that there were no significant differences among immigrants’ ethnic identity status and ethnic identification labels, *χ*^2^ (6, *N* = 460) = 10.96, *p* = .090.

### Ethnic identity development

There were no age group differences in ethnic identity status in the total sample, *χ*^2^ (6, *N* = 932) = 4.04, *p* = .672. Separate cross tabulation analyses were conducted for immigrants and natives. Chi-square analyses results indicated that there were no age group differences among immigrants’ ethnic identity status, *χ*^2^ (6, *N* = 459) = 4.96, *p* = .549. However, there were age group differences in the native group, *χ*^2^ (6, *N* = 473) = 17.03, *p* = .009, (*V* = .13). Adjusted standardized residual indicated that 12–13-year-old adolescents were more likely to be in the achieved status (*z* = 3.5, *p* < .01) and less in the moratorium (*z* = − 2.3, *p* < .05) and that 14–15-year-old adolescents were less likely to be in the achieved status (*z* = − 2.0, *p* < .05). Proportion of participants in each ethnic identity status cluster is shown in Table 1

**Table 1:** Proportion of participants (total sample, immigrants and natives) in each ethnic identity status cluster by age group

Ethnic identity status	Age groups								
	12–13 years old			14–15 years old			16–18 years old		
	Total	Immigrants	Natives	Total	Immigrants	Natives	Total	Immigrants	Natives
Achieved	32.9	34.1	31.8	26.6	36.5	18.1	29.7	39.0	16.3
Moratorium	25.8	31.8	20.8	29.8	29.1	30.3	27.1	22.7	32.7
Foreclosed	24.0	24.0	24.0	27.1	20.6	32.6	25.9	22.0	31.6
Diffused	17.3	10.1	23.4	16.6	13.8	19.0	17.1	16.3	29.4

### Ethnic identity and life satisfaction

The factorial ANOVA analyses showed that the influence of ethnic identity over life satisfaction did not differ between immigrants and natives, *F*(3, 932) = 0.85, *p* = .469. However, the influence of ethnic identity over life satisfaction was statistically significant, Welch (3, 461.58) = 22.09, *p* < .001. Games Howell post hoc multiple comparisons indicated that there were differences between all the statuses except for between the moratorium and foreclosed ones, indicating that participants in the achieved ethnic identity status were the ones who reported higher levels of life satisfaction (*M* = 3.96, SD = 0.76), followed by foreclosed (*M* = 3.77, SD = 0.88) and moratorium (*M* = 3.62, SD = 0.83) (no statistical differences between them) and finally the diffused (*M* = 3.25, SD = 1.01).

The effect was of medium size for the difference achieved vs diffused (*d* = 0.79) and foreclosed vs diffused (*d* = 0.55), and small for the difference moratorium vs diffused (*d* = 0.40), achieved vs foreclosed (*d* = 0.30) and achieved vs moratorium (*d* = 0.43).

## Discussion

### Ethnic identification

The results of this study confirm that ethnic identification is mainly determined by birthplace, for both immigrants and natives (H1). Regarding natives, although we expected that native ethnic identification be mainly determined by birthplace, it is worth mentioning that this rate was quite high (94.4%). Previous research has paid little attention to native adolescents, since ethnic identity is not supposed to be as important for natives as for ethnic minority groups. Based on this argument, maybe we could expect the marginalized identity status to be more prevalent. In the specific context we considered for our study, we think there are other additional circumstances that have been able to make native adolescents rethink their ethnic identification. These youngsters attend educational institutions and live in neighborhoods where there is a large presence of immigrants, so perhaps in ethnical homogeneous centers and neighborhoods the results would have been different. Social identity theory claims that identification with one’s own group can increase by contact with different groups, especially when they are a minority in their immediate contexts (Xu et al., 2015). In addition, this result can be a consequence of the relatively recent immigration in Spain. In other countries, like the USA, these differences have existed for a longer period and do not make mainstream adolescents redefine questions pertaining to ethnic identification.

Regarding immigrants, in accordance with previous research (Fuligni & Tsai, 2015; Fuligni et al., 2008; Kiang & Witkow, 2018), we did not find a large inclusion of concepts referring to Spain in the self-identification of immigrants born outside Spain. Despite the fact that our participants belong to the first-generation immigrants, we observed that about a quarter of them included concepts that refer to Spain in their ethnic identification, whether combined with terms that refer to their origin places or singularity. Even still, there is a minority (about 9%) who did not refer to their origin countries in their ethnic identification. This situation may be caused, to some extent, because we have studied people who emigrated while being children or adolescents (also called the 1.5 generation), which has been considered even the second generation by some authors (usually called children of immigrants, Portes, Aparicio, & Haller, 2016). This is even more apparent when individuals emigrated at a younger age, because they do not have such strong previous memories, unlike those who emigrated as adolescents or adults. Our results confirm this idea, according to previous research (Fuligni et al., 2008), because the inclusion of Spanish terms was mainly present in adolescents who emigrated at an early age (before 7 years old), confirming our second hypothesis. Young people who migrated before 7 years old can be considered members of the second generation (Berry, Phinney, Sam, & Vedder, 2006), because most of their experience has been lived in the new country. If we consider that they are similar to those who were born in the new country, then the pattern found coincides with previous research that indicates the inclusion of these terms was done by the second- and third-immigrant generations (Kiang et al., 2011).

### Ethnic identity in immigrants and natives

Our data confirmed the identity status model proposed by Marcia (1966) and founded in previous research about ethnic identity (Dimitrova, Chasiotis, Bender, & van de Vijver, 2017; Douglass & Umaña-Taylor, 2017; Yip, 2014). The results of this study confirm that immigrants more often have an achieved ethnic identity than non-immigrants (H3), coincident with previous research (Douglass & Umaña-Taylor, 2017; Kiang & Fuligni, 2009). As we have reported, immigrants were more represented in the achieved ethnic identity status (36.5%) than natives (22%), and less in the foreclosed (22% vs 29.6%) and diffused (13.5 vs 20.5%), showing they are in more advanced statuses. Solving questions regarding ethnic identity are crucial aspects for people who belong to ethnic minorities, especially for first-generation immigrants, for whom the cultural transition implies a negotiation of a new sense of belonging in the new country (Liu, 2015). They must face fundamental questions such as who they are and who they will become in the new country, while for people who belong to mainstream society this aspect is not as important for their self-concept. Finally, there were no differences in the moratorium status (around 28% where in this status in both groups), indicating adolescence is a period of exploration of ethnic identity.

On the other hand, the relationship between ethnic identity labels used by immigrants and ethnic identity status was not confirmed (H4). In this way, we did not find that youths who identified only with their culture of origin show a higher level of achieved identity as expected from previous research. Authors such as Fuligni et al. (2008) indicated that retaining national labels could require more effort, and this could reflect on higher identity exploration and commitment levels.

However, our results show that immigrant adolescents in Spain are not just accepting labels without exploration and commitment. That could be explained by different factors. First, contrary to most research where they include different generations, all participants in this study were first-generation immigrants, so when they use an ethnic label that refers to the host country, it could imply a deep process of rethinking their ethnic identity. Second, in Spain, official forms in which people are categorized based on their ethnicity are not commonly used as in other countries, like the USA or the UK. Our participants’ lack of familiarity with categorizing themselves could imply an active process of exploration and commitment when they choose to use labels in the present study.

### Ethnic identity development

The results of this study do not confirm that older adolescents have a higher degree of achieved ethnic identity (H5), as was expected (Syed, 2015). Moreover, our results show different patterns in ethnic identity development across adolescence for immigrants and natives, and that the development of an achieved ethnic identity is not complete by the end of adolescence. Regarding immigrants, ethnic identity shows the same pattern in all age groups; ethnic status was stable across the three age groups (from youngest to oldest): in the first place achievement (34, 37, and 39%), in the second moratorium (32, 29, and 23%), in the third foreclosed (24, 21, and 22%), and lastly in the diffused (10, 14, and 16%). These results could indicate that immigrants have not yet finished completing their ethnic identity development. Because we have focused on the first generation and in a county with relatively recent ethnic and racial diversity, people who come from other countries may not have the opportunity to develop their ethnic identities during adolescence, but they are forced to develop them from the moment they become aware of these aspects. Ethnic identity may be more important for them and becomes a key factor to understand who they are, in comparison with ethnic minority adolescents who live in a country with a long tradition of immigration, where cultural diversity has always been present, so that they do not feel so different or feel forced to develop an ethnic identity quickly.

On the other hand, the study of natives’ ethnic status shows that there is a progressive decrease of native participants in the achieved status across age groups (from youngest to oldest: 32, 18, and 16%), with higher representation in the other statuses. This may suggest that native adolescents initially identify with their nationality in opposition to other ethnicities, without any developmental process. When the developmental process begins, they start to question their ethnic identity and do not feel so sure about it, increasing the exploration about the meaning of their ethnic identity.

### Ethnic identity and life satisfaction

Finally, the results of this study support partially the last hypothesis (H6); although ethnic identity was positively related to life satisfaction for both immigrants and non-immigrants, this relation was not stronger in the case of immigrants. Our results showed that an achieved ethnic identity has a positive influence on life satisfaction, which matches previous research (Abubakar et al., 2014; Adams et al., 2016; Fuligni et al., 2008). However, most of the research has included only immigrant or ethnic minority adolescents, taking for granted that ethnic identity is not important for mainstream adolescents’ life satisfaction. Taking into consideration that ethnic identity is more important for immigrants than for non-immigrants (or minority groups compared to the majority), it would be expected that ethnic identity has more influence on immigrants’ life satisfaction. However, our results support that having an achieved ethnic identity is related with higher levels of life satisfaction, for both natives and immigrants, supporting the idea that ethnic identity is important for mainstream adolescents too, especially in settings where they are exposed to multicultural contexts.

### Limitations and future research

This study presents some limitations that are necessary to mention and that could offer guidelines for future research. One of the limitations of this study is that is a cross-sectional study, although it allows us to study differences between age groups, a longitudinal study could provide more information about the development of ethnic identity at an individual level. Kiang et al. (2010), who also did not find differences between age groups in ethnic identity, pointed out that their results do not contradict an identity increment hypothesis throughout adolescence, because individuals change during their lifetime. A longitudinal study is necessary to confirm it could apply the same to immigrant adolescents in Spain.

Another limitation is based on the compositions of schools that where multiethnic. We believed some of our finding, as we have discussed, relied on the specific context of our research which have been carried out. Future research could compare if ethnic identity remains important for native adolescents in Spain in a context where there is no interaction with ethnic minorities. However, in Spain, as well as in most parts of the world, cultural diversity is increasing, so we recommend focusing on multiethnic context, because no interaction between majority and minority ethnic groups is going to turn into an exception.

The last limitation to mention was that the instruments were in Spanish, although prior to data collection we consulted in schools about the necessity of translations to other languages and they informed all immigrants who have a proper level of Spanish, 10 participants refuse to participate alluding they did not have a high enough level to respond properly. We cannot be sure if maybe there are other reasons, but future research could have translations of the questionnaires for these cases.

## Conclusions

The current study offers important evidence regarding ethnic self-identification, ethnic identity, and life satisfaction of immigrants and native adolescents living in Spain, contributing to the generalization of knowledge regarding ethnic identity to other cultural contexts.

Although we found that most immigrants identify with their country of birth, about a quarter also included references to Spain (18.2% in conjunction and 9.2% alone). These results indicate that, although country of origin is important for immigrants’ ethnic self-identification, they are not directly equivalent. Despite these differences in adolescents’ ethnic identification, research often tends to describe participants ethnicity based on country of birth (or even country of birth of their parents). The results of this study contribute to the claim that country of origin should not be used to determine participants’ ethnicity (Parameshwaran & Engzell, 2015).

In our study, contrary to most literature available that focus on ethnic identity, we have included native adolescents and not just immigrant adolescents. The results show that ethnicity turns into an important matter also for natives, at least in the context of multiethnic contact. A sense of belonging in terms of ethnic groups is necessary both for immigrants and native who live together, as an important part of the identities they are developing. Who they are, and how they relate to others in term of ethnic groups become relevant questions. Our results indicate that the development of an achieved ethnic identity is not complete by the end of adolescence, especially in multiethnic contexts, with different patterns for immigrants and natives; showing there were no differences between age groups among immigrants and there where a decrease in achieved ethnic identity in natives.

Finally, in accordance with previous research, the present study also shows that solving these questions is more important for immigrants, who develop a stronger sense of ethnic identity (achieved ethnic identity) than non-immigrants, even though having a strong sense of ethnic belonging is related with life satisfaction for both immigrants and natives.

## Author’s information

Laura Lara holds a Ph. D. in Psychology from the University of Seville (Spain) and is currently a professor and researcher at the Universidad Autonoma de Chile, where she teaches in the department of developmental psychology, as well as participating in research projects relating to aspects of human development and education.
